# Neurotypicals with higher autistic traits have delayed visual processing of an approaching life-sized avatar’s gait: an event-related potentials study

**DOI:** 10.3389/fnhum.2023.1113362

**Published:** 2023-04-20

**Authors:** Ryo Inokuchi, Hiroko Ichikawa, Masataka Yamamoto, Hiroshi Takemura

**Affiliations:** ^1^Department of Mechanical and Aerospace Engineering, Tokyo University of Science, Chiba, Japan; ^2^Institute of Arts and Sciences, Tokyo University of Science, Chiba, Japan

**Keywords:** autism spectrum disorder (ASD), higher autistic traits, event-related potentials, visual motion processing, approaching life-size avatar

## Abstract

**Introduction:**

Autism spectrum disorder (ASD) is a neurodevelopmental disorder, which is reportedly related to difficulties in the visual processing of human motion, such as biological motion and gestures. Moreover, neurotypical (here, we mention it as individuals without a diagnosis) adults with autistic traits are clumsier than those without autistic traits when passing by others. It is still unclear whether the clumsiness derived from atypical visual processing of another’s approaching gait motion. We aim to address this question by investigating the association between autistic traits in neurotypical adults and the visual processing of an approaching life-sized avatar’s gait.

**Methods:**

We clarified a typical visual motion processing and autistic traits in daily life in 26 neurotypical adults by analyzing the subthreshold autism trait questionnaire (SATQ) score, a 24-item self-report scale of ASD, and event-related potentials (ERPs) in response to walking motion of a passing avatar. Videos of walking life-sized virtual avatars approaching and retreating were presented as visual stimuli.

**Results and discussion:**

The association between the participants’ SATQ scores and the latencies and amplitudes of the ERPs was examined. ERP components (N170 and P200) components were identified at T5 and T6 electrodes. Participants reporting higher SATQ scores had longer latencies of P200 at T6 and lower amplitudes of P200 at T5 and T6 electrodes for the approaching avatar than those reporting lower SATQ scores. These findings indicate that adults with autistic traits have delayed and less sensitive visual processing of the approaching avatar. It suggests that while passing another person, these individuals have atypical visual processing of their approach. This study may contribute to elucidating autistic traits from the perspective of visual processing in an environment simulating daily life.

## 1. Introduction

Autism spectrum disorder (ASD) is a neurodevelopmental disorder characterized by two distinct features: (a) persistent deficits in social communication and interaction across multiple contexts; and (b) restricted, repetitive patterns of behavior, interests, or activities (Frith and Happé, 2005; Walter et al., 2009; American Psychiatric Association [APA], 2013; Lord et al., 2018). Social interaction deficits observed in individuals with autistic traits also include difficulty in maintaining interpersonal relationships and understanding the expressions and gestures of others (Vukusic et al., 2017; Lord et al., 2018). Successful nonverbal communication requires appropriate processing of visual information. Individuals with a diagnosis of ASD showed difficulties in emotion recognition in comparison to typically developing individuals, which may be due to reduced gaze at another person’s eyes (Klin et al., 2002; Nakano et al., 2010) and mouth (Pelphrey et al., 2002). The most informative feature of fear appears around the mouth and thus individuals with a diagnosis of ASD find it difficult to recognize fearful facial expressions (Pelphrey et al., 2002). In the case of understanding another person’s intention by observing his/her physical gestures, such as walking past another person, one can successfully avoid collision only if one perceives the other person’s approach without delay. Atypical visual processing could encumber social interactions for individuals with autistic traits. It is necessary to focus on an understanding of visual motion processing during interactions with others to better elucidate the atypicality of social interactions in individuals with autistic traits. Previous studies reported the brain-behavior relationship of visual motion perception. Schallmo et al. (2019) measured functional magnetic resonance imaging (MRI) responses in the motion-selective region middle temporal (MT) and simultaneously tested the participant’s visual motion perception. They revealed that the level of glutamate, an excitatory neurotransmitter, in the MT region was associated with both stronger BOLD signals and better motion perception. The imbalance of excitatory/inhibitory transmission has been observed in individuals with a diagnosis of ASD (Bernardi et al., 2011) and modulated the perceived visual motion (i.e., Takeuchi et al., 2017). A previous study has shown that individuals with autistic traits have awkward physical motion in situations where another person is approaching. Shigeta et al. (2018) focused on gait characteristics in neurotypical (in this paper, we mention it as individuals without a diagnosis) adults with autistic traits and measured participants’ physical motion using a motion capture system during participants were walking past each other. The severity of autistic traits of their participants were assessed by conducting the questionnaire, the subthreshold autism trait questionnaire (SATQ) a 24-item self-report scale of ASD. They reported that participants reporting higher SATQ scores had larger standard deviations from the norm of angular velocity in the waist than those reporting lower SATQ scores (Shigeta et al., 2018). The findings suggest that waist movements while passing another person were positively correlated with the score of SATQ. Since awkward waist movements would be induced in parallel with delayed physical responses to avoid a collision, it is suggested that individuals with autistic traits have atypicality in visually processing another person’s approach.

Event-related potentials (ERPs) have been used to evaluate brain function and clarify the mechanisms underlying various neurological disorders because ERPs appear in response to perceptual processing and reflect visual information processing (Tobimatsu and Celesia, 2006; Horvath et al., 2018). Previous studies have employed ERPs to understand visual motion processing in individual with a diagnosis of ASD (Jeste and Nelson, 2009; Yamasaki et al., 2011). Yamasaki et al. (2011) measured ERP response to visual stimuli, including radial and horizontal motion, from the adult individuals with a diagnosis of ASD and neurotypical adults. Radial motion consisted of optic flow containing dots moving radially outward, and horizontal motion consisted of dots moving to the right. The results showed that the latencies of both N170 and P200 to radial optic flow were significantly prolonged in individuals with ASD compared to neurotypical adults and the latencies of ERPs to the horizontal motion were not prolonged in either group. Other studies have measured ERPs for a point-light walker (PLW) that simulated an approaching person and compared the latencies of the ERPs between the group of neurotypical adults with higher SATQ scores with the group with lower SATQ scores. PLW is a visual stimulus consisting of moving dots that record joint positions during human body movement. The results showed that the latency of N1 observed at the P8 electrode placed according to the 10–10 system (that is, corresponding to the T6 electrode placed according to the 10–20 system) was longer in the group with higher SATQ scores than that with lower SATQ scores (Ichikawa et al., 2019; Inokuchi et al., 2022a). These studies indicate that visual processing of expansion movements is atypical in individuals with autistic traits. Most studies on visual motion processing associated autistic traits used moving dot stimuli, such as radial optic flow, which represents non-biological motion (Yamasaki et al., 2011) or an approaching PLW (Ichikawa et al., 2019; Inokuchi et al., 2022a). However, the visual processing of natural and smooth gait motion by a more realistic avatar remains unclear in individuals with autistic traits. Although using the recordings of an actual human gait seem to be better to elucidate the atypical visual processing showed in real life than those in radial optic flow or PLW, it contains task-irrelevant factors that affect visual processing, such as the avatar’s facial information (i.e., gaze shift, gaze direction, facial expression, facial attractiveness, and asymmetric gait traits of an actual person). Therefore, in this study, the video of a life-size avatar approaching was used as a visual stimulus. Measuring the neural responses to gait motion displayed by a realistic avatar would provide important clues for determining the behavioral characteristics of ASD in daily life.

Herein, we investigated the association between autistic traits and visual processing of another person’s approach by measuring the ERPs for videos using a life-sized virtual avatar. In this study, we focused on autistic traits exhibited by individuals who had not been diagnosed with ASD. This is because autistic trait is observed as a continuum without boundaries whose symptoms vary widely in severity, and some individuals have autistic traits even without ASD diagnosis (Mukaetova-Ladinska et al., 2012). Numerous individuals with autistic traits remain undiagnosed and are referred to as the broader autism phenotype (BAP) (Piven et al., 1997). Many survive childhood and adulthood and escape the diagnosis of ASD often with family support or by working in a supportive environment. A recent study reported that children with BAP experience difficulties in some domains of visual perception (Sabatino DiCriscio and Troiani, 2018). More studies focusing on the relation between autistic traits exhibited by undiagnosed individuals and visual processing in adults are needed. Therefore, the present study recruited adult undiagnosed individuals and examined the association between their severity of autistic traits and visual processing. Since the severity of autistic traits is a continuum, as mentioned above, we do not have the unique numerical criteria to determine if the person reporting the SATQ score is ASD or BAP. In the experiment, participants viewed three different patterns of avatar movements: approaching walking movement (AW), receding walking movement (RW), and standing (ST). The latency and amplitude of N170 and P200 at T5 and T6 electrodes placed in accordance with the 10–20 system were identified, and the association with autistic traits for the more realistic biological motion were examined.

## 2. Materials and methods

### 2.1. Participants

This study enrolled 30 neurotypical male university students, with a mean (± standard deviation) age of 22.17 (± 1.19) years. The study participants were neurotypical males, who had never been diagnosed with ASD, consistent with a previous study (Shigeta et al., 2018) that suggested an association between atypical visual processing of another individual’s gait motion and autistic severity. Confounding factors, such as comorbidities and autism-related symptoms, were not considered. We recruited only male participants for this study due to the university male-to-female ratio was quite unbalanced.

The participants completed the SATQ, a 24-item self-report instrument that quantitatively measures autistic traits, with each question rated on a 4-point Likert scale ranging from “false, not at all” to “very true” (Nishiyama et al., 2014). The SATQ is especially sensitive to individuals who are not clinically diagnosed with ASD but show subliminal autistic traits. The SATQ score was calculated by summing the scores for the 24 items. A higher SATQ score indicates higher autistic traits. The participants’ mean ± standard deviation SATQ score was 27.77 ± 8.02 out of a total of 72 points.

Informed consent was obtained from each participant before the experiment. The experimental protocol was approved by the Ethics Committee of the Tokyo University of Science (approval number: 16027).

### 2.2. Visual stimuli and procedure

**FIGURE 1:**
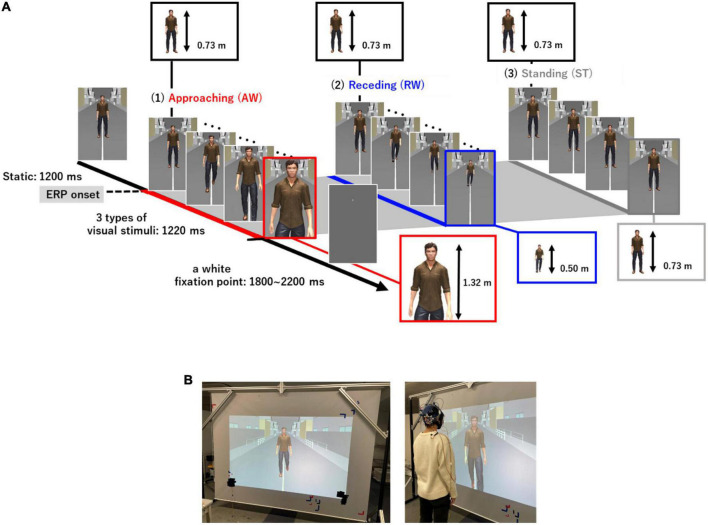
**(A)** Three types of visual motion stimuli and the experimental procedure in one trial. The standing image was presented for 1,200 ms, and thereafter, three types of visual stimuli (AW, RW, ST) were presented for 1,220 ms each. After the end of the video for each condition, a white gazing point was displayed for 1,800–2,200 ms. The onset time of each visual stimulus (AW, RW, ST) was used as the ERP onset. The actual height taken up by the virtual avatar in the visual field of the participants changed from 0.73 to 1.42 m in the AW condition and from 0.73 to 0.50 m in the RW condition. In the ST condition, the size of the avatar remained at 0.73 m. Moreover, the maximum avatar size in the AW condition, if it had been projected onto a large enough screen, would have been 2.3 m. **(B)** Experimental environment. Visual stimuli were cast onto a 2.7 m wide and 1.9 m high screen using a projector with a refresh rate of 60 Hz. The participants observed the visual stimuli in the standing position at 0.6 m away from the screen. AW, approaching walking movement; RW, receding walking movement; ST, standing; ERP, event-related potential.

We created visual stimuli using a life-sized virtual avatar to imitate a more realistic setting of “passing others” compared to previous studies (Figure 1). The Unity 3D game engine (version 2020.2.3f1, Unity Software Inc., San Francisco, USA) was used to create the visual stimuli. The visual stimuli consisted of a virtual avatar and a background that imitated a typical street scene. Three different patterns of avatar movement were created [AW movement, RW movement, and ST (not walking)]. In the AW condition, the video depicted the virtual avatar approaching the participant and the visual angle of the height of the person changed from 62.4 to 95.6. In the RW condition, the video showed a person moving away from the participant and the visual angle of the height of the person changed from 62.4 to 45.2. We included the RW condition in addition to the AW condition to examine if the prolonged ERP latency observed for PLW depended on the direction in which the avatar walked. Thus, in the experiment, two types of conditions were used as the target stimuli. The third condition ST depicted a video of a person swaying from side to side and standing in place. The ST condition was presented as a non-target stimulus to prevent participants from predicting the avatar’s movements and prevent them from preparing to press the button in the attention task. Figure 1 shows the three types of visual motion stimuli and the experimental procedure of one trial. First, a still image of the virtual avatar standing was presented for 1,200 ms for all visual stimuli. Thereafter, one of three (AW, RW, and ST) visual motion stimuli were initiated, which lasted for 1,220 ms each. After the video for each condition ended, a white fixation point was displayed for 1,800–2,200 ms. Each trial was presented for 4,220–4,620 ms. Five sets of visual stimuli were presented and each set consisting of 60 trials. Therefore, the participants randomly observed the AW and RW videos for 120 trials and the ST video for 60 trials. Participants were given a break between sets to prevent fatigue from affecting the results of electroencephalography (EEG) (visual stimulus video, Supplementary material).

The visual stimuli used in the experiment were cast onto a 2.7 m wide and 1.9 m high screen using a projector with a refresh rate of 60 Hz. Participants stood 0.6 m away from the screen and were instructed to focus on a fixation point at the center of the stimulus to adjust the height of the screen.

### 2.3. Attention task

During the EEG recording, participants performed the button-pressing task whose purpose was to get the participants to concentrate on the visual stimuli. During this task, participants were instructed to determine the direction in which the virtual avatar on the screen moved and answer using a numeric keypad. The participants were instructed to press “left” (4 on the numeric keypad) if they judged that the virtual avatar moved to the left from their perspective and to press “right” (6 on the numeric keypad) if they judged that the virtual avatar loved to the right, as quickly as possible. The participants wore the numeric keypad, which was fixed around their thighs with a belt, on the dominant side. The numeric keypad was positioned so that participants could press the buttons while standing. Participants were allowed to answer intuitively to ensure that they could respond to as many trials as possible. Since the task was performed such that the participants’ concentration was maintained during the experiment, the analysis of their performance in the additional task is beyond the scope of this study and has not been mentioned here. Moreover, the response rate during the AW+RW trials was calculated as the number of key responses by the participant divided by the total number of trials that could be recorded. The response rate during AW+RW trials was almost 1. These results indicated that the participants successfully paid attention to the screen due to the attention task.

### 2.4. EEG recording

Electroencephalography (EEG) and electrooculography (EOG) measurements were acquired using a simple 8-channel electroencephalograph (Ultracortex “Mark IV” EEG Headset; OPENBCI). The active dry electrodes were placed at six locations (C3, C4, T5, T6, O1, and O2) according to the 10–20 system. Two other electrodes were placed above the right eyebrow and below the right eye to measure the EOG signal. The EOG data were used to detect artifacts from eye movements and blinking. The reference marker was placed on the tip of the nose, far from the temporal area. A ground electrode was placed over the right earlobe. EEG and EOG were performed at a sampling frequency of 250 Hz.

### 2.5. ERP analysis

**FIGURE 2:**
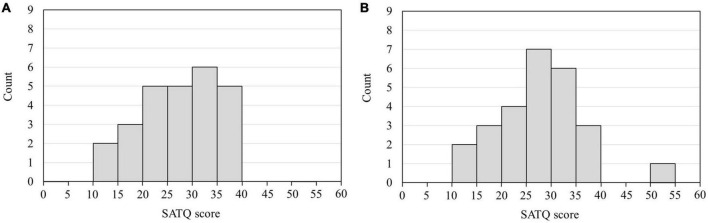
Histogram of the SATQ scores of 26 participants used in the ERP analysis. The horizontal axis is the SATQ score of each participant, and the vertical axis is the number of participants. **(A)** SATQ scores of 26 individuals were used to analyze ERPs at the T5 electrode. Four participants whose ERPs could not be identified at the T5 electrode were excluded from the analysis. The mean and standard deviation of the SATQ score in the participants was 26.85 ± 7.21 out of 72 points. **(B)** SATQ scores of 26 individuals were used to analyze ERPs at the T6 electrode. Four participants whose ERPs could not be identified at the T6 electrode were excluded from the analysis. The mean and standard deviation of the participants’ SATQ score was 27.04 ± 8.13 of 72 points. SATQ, subthreshold autism trait questionnaire; ERP, event-related potential.

The frequency of the bandpass filter ranged from 1 to 20 Hz. After filtering, EEG data were divided into epochs from 200 ms before to 600 ms after the onset of each visual stimulus (AW, RW, and ST). Non-neural artifacts, such as blinking and eye movements, were rejected by visual inspection and independent component analysis. Subsequently, the ERP waveforms were calculated by averaging all epochs, except the artifacts. EEG data from 200 ms before each visual stimulus (AW, RW, and ST) were used as the baseline. The ERP components in each channel for each participant were identified from the grand-average waveform by visual inspection. In this study, we identified the ERP component N170, which appears as a negative peak around 170 ms from the onset, and the ERP component P200, which appears as a positive peak around 200 ms from the onset. Participants and electrodes with strong noise were excluded from the analysis. The N170 and P200 at the T5 and T6 electrodes were used as evaluation indicators because this study focused on the findings of Ichikawa et al. (2019) and Inokuchi et al. (2022a), i.e., “individuals with higher SATQ scores traits have delayed ERP (N1) latency of T5 and T6 electrodes for approaching PLW than individuals with lower ASD traits,” and the N170 and P200 components were observed at the T5 and T6 electrodes in this study. Therefore, from the outset, the analysis focused on the ERPs observed at the T5 and T6 electrodes. The data of participants whose ERPs at the T5 and T6 electrodes were not identified were excluded from the analysis. Thus, ERP data from four participants were excluded at both electrodes, and data from 26 participants were used for analyses at the T5 and T6 electrodes. Figure 2 depicts the histograms of the SATQ scores of the 26 participants included in the ERP analysis at each of the T5 and T6 electrodes. The acquired data were pre-processed using EEGLAB (MATLAB, MathWorks, MA, USA)^1^ (Delorme and Makeig, 2004).

### 2.6. Statistical analysis

We conducted multiple regression analysis with the exhaustive search (ES) method to select effective variables to explain the SATQ score. The ES method of variable selection, in which the coefficient of each variable is 0 or other, entails exhaustively searching 2^*N*^−1 = _*N*_*C*_1 *N*_*C*_2_ ⋯ _*N*_*C*_*K*_ ⋯ _*N*_*C*_*N*_ models, where N is the number of variables (Ichikawa et al., 2014; Nagata et al., 2015; Igarashi et al., 2018). Previous studies on the ES method have shown that it is the only algorithm that can find the optimal subset of feature variables, since it explores the entire solution space (Cover and Van Campenhout, 1977; Igarashi et al., 2016). The ES method is also used for assessment in studies performing multichannel functional continuous-wave near-infrared spectroscopy measurements and is suitable for evaluating brain activity (Ichikawa et al., 2014). In this study, multiple regression analysis using the ES method was performed at the T5 and T6 electrodes, respectively. We used the SATQ scores of each participant as the objective variables. Eight ERP indicators at electrode T5 ([latencies and amplitudes] × [N170 and P200t] × [in AW and RW conditions]) were used as the explanatory variables while analyzing the T5 electrode. Therefore, 255 different models (2^8^−1) were exhaustively searched for the T5 electrode. The data obtained at the T6 electrode were analyzed in a similar manner.

Thereafter, the predictive accuracy of each multiple regression model was examined using cross-validation. For cross-validation, the dataset was split into training and testing sets. We used leave-one-out cross-validation (LOOCV), which is effective when the number of samples is small. LOOCV entails designating only one instance from the entire dataset as the test data, followed by swapping the test data and training data, and repeating the process for validation until all cases have been examined as the test data. The following procedure was used for LOOCV or the data of the 26 participants with identified ERPs. First, we defined one dataset (e.g., participant 1′s data) as the test data, and the remaining 25 (i.e., data of participants 2 to 26) as the training data. Multiple regression analysis was conducted using the training data and a regression model was obtained. The regression model was then applied to the test data, and the cross-validation error (CVE) was calculated. Next, we defined other data (e.g., participant 2′s data) as the test data, and the remaining 25 (i.e., the data of participant 1 and participant 3 to participant 26) as the training data, conducted regression analysis and cross-validation and calculated the CVE. The procedure was repeated 26 times, and the CVE for the regression model was obtained by averaging the 26 CVEs. In addition, to compare the impact of each explanatory variable on the SATQ score, standardized partial regression coefficients were calculated for each explanatory variable for each model, which are shown in the color map within the weight diagram. In the standardized partial regression coefficient, the objective variable and each explanatory variable are standardized to mean = 0 and variance = 1. In the weight diagram (Figures 4, 5), a color map shows the value of the standardized partial regression coefficient for each explanatory function included in the model. In accordance with the color bar, red and yellow indicate positive values, and blue indicates negative values.

Herein, CVE was defined as the mean absolute error (MAE) between the SATQ score predicted by the model obtained from multiple regression analysis and the actual SATQ score. The formula for calculating the MAE is shown in Equation 1. The advantage of the MAE is that it is less susceptible to outliers than the root mean square error or mean squared error. To evaluate the effective variables that could explain SATQ scores, we created a weight diagram plot using the CVE and standardized partial regression coefficient in each model. All statistical analyses were conducted using MATLAB version 2021a (MathWorks, Inc.).


(1) $$M\,A\,E\,\left(m\,e\,a\,n\,a\,b\,s\,o\,l\,u\,t\,e\,e\,r\,r\,o\,r\right)=\frac{1}{n}\,\sum_{i=1}^{n}\left|{\hat{y}}_{i}-y_{i}\right|$$

where, ${\hat{y}}_{i}$ is the SATQ score predicted by the model, *y*_*i*_ is the actual SATQ score, and ***n*** is the number of samples (*n* = 26).

In this study, we also analyzed 16 variables of the ERP indicator using other variable selection methods, such as elastic net (Zou and Hastie, 2005) and lasso (Tibshirani, 1996). However, the results showed that no explanatory functions were selected and only the constant terms remained, so the ERP data could not be interpreted well. Therefore, the ES method was used in this study, which facilitates exhaustive analysis of all multiple regression models.

## 3. Results

### 3.1. Grand-average waveform

**FIGURE 3:**
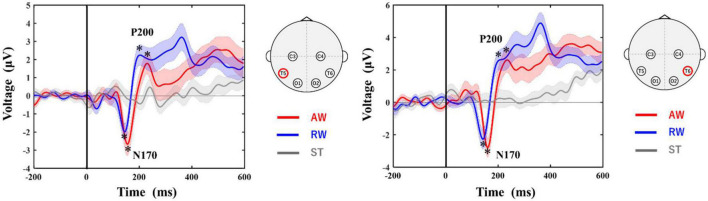
Grand-averaged ERP waveforms at T5 and T6 electrodes. The horizontal axis shows the latency from the onset. The vertical line at time 0 indicates the time point when the walker started to approach/recede. The vertical axis shows the ERP potential. The red line represents ERPs in the AW condition, the blue line represents ERPs in the RW condition, and the gray line represents ERPs in the ST condition. The asterisks in the figure indicate the peak of each ERP (N170, P200) identified in this study. The shaded area around each ERP trace indicates the standard error (SE) of the mean. AW, approaching walking movement; RW, receding walking movement; ST, standing; ERP, event-related potential. *Indicates the selected peak of each ERP (N170, P200).

Figure 3 shows the grand-average waveforms at the T5 and T6 electrodes. The negative ERP component N170 around 170 ms and the positive ERP component P200 around 200 ms appeared in both the AW and RW conditions. However, neither the N170 nor the P200 component appeared in the ST condition, in which the avatar was standing upright on the screen.

### 3.2. Multiple regression analysis

Multiple regression analysis with the ES method was performed using the amplitude and latency of each ERP at the T5 and T6 electrodes. LOOCV was performed to evaluate the accuracy of each model.

**FIGURE 4:**
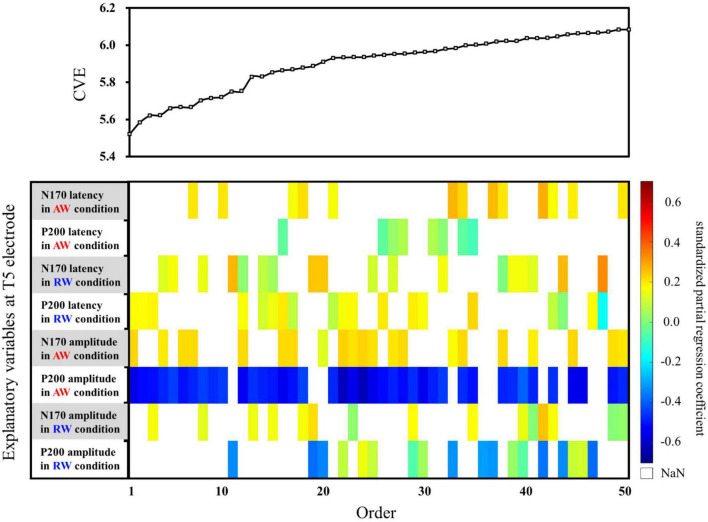
Graph showing the values of the cross-validation errors (upper panel) and weight diagram plots (lower panel) for each model when performing multiple regression analysis with ES. The SATQ scores of each participant were designated as the objective variable and eight ERP indicators at electrodes T5 [(latencies and amplitudes) × (N170 and P200) × (in AW and RW conditions)] were the explanatory variables. Fifty of the 255 different regression models are arranged along the horizontal axis based on the model with the smallest CVE. The vertical axis presents the eight explanatory variables. The color map shows the value of the standardized partial regression coefficient for each explanatory function included in the model. The red/yellow spectrum indicates positive values, and the blue spectrum indicates negative values. White areas in the color map indicate that the explanatory variable is not included in the model. At the T5 electrode, “P200 amplitude in AW condition” (third row from the bottom) was the most frequently selected explanatory variable in 40 of the 50 models. The second most frequently selected variable was “N170 amplitude in the AW condition” (fourth row from the bottom), which was selected 21 times among the 50 models. AW, approaching walking movement; RW, receding walking movement; ST, standing; SATQ, subthreshold autism trait questionnaire; ERP, event-related potential; ES, exhaustive search.

First, multiple regression analysis was conducted with eight ERP indicators (N170 and P200 latencies and amplitudes under AW and RW conditions) at the T5 electrode (Figure 4). The upper panel of Figure 4 depicts the CVE results for each model as an evaluation indicator of prediction accuracy. Fifty of the 255 models were arranged along the horizontal axis based on the model with the smallest CVE. The lower panel in Figure 4 depicts a weight diagram plot with ES. Each of the 255 models generated using multiple regression analyses was evaluated using the CVE. White areas in the color map indicate that the explanatory variable is not included in the model. “P200 amplitude in the AW condition” was selected in 40 of the 50 multiple regression models with a small CVE, making it the most frequently chosen variable from among the eight explanatory variables. In addition, the color map in the weight diagram shows the value of the standardized partial regression coefficient for each explanatory function included in the model. The magnitude of the standardized partial regression coefficients in each explanatory variable can be interpreted as follows: when the coefficient has a positive value, the value of the explanatory variable is higher, i.e., a higher SATQ score for the objective function. A negative coefficient value implies a smaller value of the explanatory variable, i.e., a lower SATQ score. The value of the standardized partial regression coefficients for “P200 amplitude in the AW condition” were negative for all 40 selected models. Additionally, the second most frequently selected variable was “N170 amplitude in the AW condition,” which was selected 21 times in the 50 models. Table 1 shows the multiple regression model with the smallest CVE at the T5 electrode. Three variables were entered as predictors: “P200 latency in RW condition,” “N170 amplitude in AW condition,” and “P200 amplitude in AW condition.” The multiple regression model was statistically significant [*F*(3,22) = 3.094, *p* = 0.048, adjusted *R*^2^ = 0.200]. Single variable regression with the SATQ score and “P200 amplitude in AW condition,” the most frequently selected explanatory function at the T6 electrode, was performed as an additional analysis to confirm the direction of influence. Thus, similar to the weighted diagram plot, “P200 amplitude in AW condition” was found to be negatively related to the SATQ score (standardized partial regression coefficient=−0.47).

**TABLE 1:** Multiple regression analysis table of the model with the smallest CVE at the T5 electrode.

	Unstandardized coefficients		Standardized coefficients	
**Variable at T5**	***B***	**Standard error**	**Beta**	***P*-value**
P200 latency in the RW condition	0.04	0.04	0.18	0.35
N170 amplitude in the AW condition	0.59	0.48	0.22	0.23
P200 amplitude in the AW condition	−1.24	0.42	−0.53	0.01*

Adjusted *R*^2^ = 0.20, *indicates *p* < 0.05. CVE, cross-validation error; AW, approaching walking movement; RW, receding walking movement.

**FIGURE 5:**
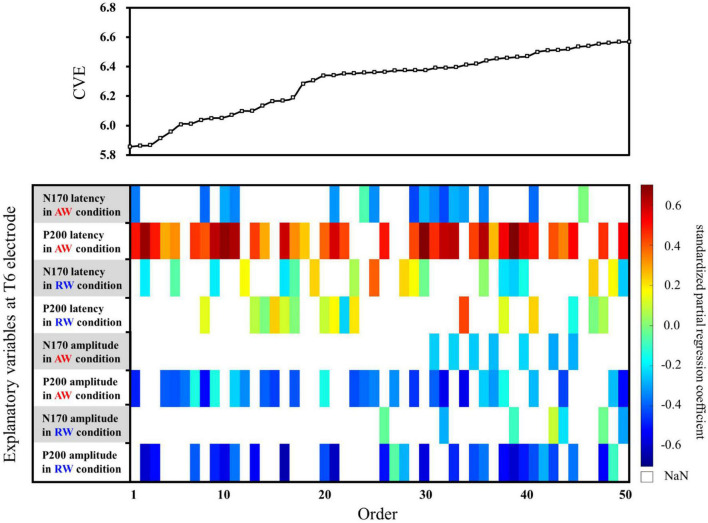
Graph showing the values of the cross-validation errors (upper panel) and weight diagram plots (lower panel) for each model when performing multiple regression analysis with ES. The SATQ scores of each participant were the objective variables and eight ERP indicators at electrodes T6 [(latencies and amplitudes) × (N170 and P200) × (in AW and RW conditions)] were the explanatory variables. Fifty of the 255 different models are arranged along the horizontal axis based on the model with the smallest CVE. The vertical axis presents the eight explanatory variables. At the T6 electrode, “P200 latency in AW condition” (second row from the top) was the most frequently selected explanatory variable in 36 of the 50 models. The second most frequently selected variable was “P200 amplitude in the AW condition” (third row from the bottom), which was selected 28 times from among the 50 models. AW, approaching walking movement; RW, receding walking movement; ST, standing; SATQ, subthreshold autism trait questionnaire; ERP, event-related potential; ES, exhaustive search.

Second, multiple regression analysis was performed using eight ERP indicators at the T6 electrode. The weight diagram plot with ES is shown in Figure 5, where “P200 latency in the AW condition” was the most frequently selected explanatory variable of the eight explanatory variables, i.e., it was selected in 36 of the 50 multiple regression models. The value of the standardized partial regression coefficients for “P200 latency in the AW condition” were positive for all 36 models selected. The second most frequently selected variable was “P200 amplitude in the AW condition,” which was selected 28 times in the 50 models. Table 2 shows the multiple regression model with the smallest CVE at the T6 electrode. Three variables were entered as predictors: “N170 latency in AW condition,” “P200 latency in AW condition,” and “P200 amplitude in AW condition.” The multiple regression model was statistically significant [*F*(3,22) = 3.135, *p* = 0.046, adjusted *R*^2^ = 0.204]. Single variable regression with the SATQ score and “P200 latency in AW condition,” the most frequently selected explanatory function at the T6 electrode, was performed as an additional analysis. Thus, similar to the weighted diagram plot, “P200 latency in AW condition” was found to be positively related to the SATQ score (standardized partial regression coefficient = 0.24).

**TABLE 2:** Multiple regression analysis table of the model with the smallest CVE at the T6 electrode.

	Unstandardized coefficients		Standardized coefficients	
**Variable at T6**	***B***	**Standard error**	**Beta**	***P*-value**
N170 latency in the AW condition	−0.19	0.12	−0.36	0.12
P200 latency in the AW condition	0.16	0.07	0.50	0.04*
P200 amplitude in the AW condition	−1.06	0.41	−0.49	0.02*

Adjusted *R*^2^ = 0.20, * indicates *p* < 0.05. CVE, cross-validation error; AW, approaching walking movement; RW, receding walking movement.

## 4. Discussion

We investigated the association between autistic traits and visual processing of another person’s approach by measuring the ERPs for videos depicting a walking life-sized virtual avatar. Two ERP components, N170 and P200, were identified for each participant in the AW and RW conditions, respectively. The optimal multiple regression model was exhaustively searched, respectively, on the data obtained at the T5 and T6 electrodes, among the possible subsets containing eight variables, those were the latency and amplitude of N170 and P200 in the AW and RW conditions. The results showed that “P200 amplitude in the AW condition” was the most frequently selected variable among the top 50 models at the T5 electrode, and “P200 latency in the AW condition” was the most frequently selected variable at the T6 electrode. In the regression model containing these variables as predictor, the standard partial regression coefficient for “P200 amplitude in the AW condition” at the T5 electrode showed a tendency to adopt negative values for the selected models in the weight diagram. The standard partial regression coefficients for “P200 latency in the AW conditions” at the T6 electrode showed a tendency to adopt positive values. These results indicated that the longer the P200 latency for the AW condition at the T6 electrode and the smaller the P200 amplitude for the AW condition at the T5 electrode, the higher the participant’s SATQ score. These findings indicated that the P200 latency observed at the T6 electrode and P200 amplitude observed at the T5 and T6 electrode in response to watching a video of an approaching person could explain the magnitude of autistic traits. It would be suggested that individuals with autistic traits have more delayed processing of another person’s approach. Such the association between the severity of the autistic trait and prolonged latency of P200 is consistent with the previous study that showed that individuals with diagnosis of ASD have longer latencies of N170 and P200 to radial optical flow (Yamasaki et al., 2011). Furthermore, this is the first study that identified negative associations between P200 amplitude observed at T5 and T6 electrode and autistic traits, which may reflect difficulties in early visual processing in individuals with autistic traits.

The association between autistic traits and the visual processing of an approaching avatar shown in this study may be related to the atypical gait characteristics suggested in a previous study (Shigeta et al., 2018). That study suggested that individuals with autistic traits have atypical gait, in which they approach their counterparts more closely and twist their waist greatly to avoid a collision when walking past each other. Appropriate visuomotor control is required to assume appropriate motion patterns in response to obstacles in situations such as walking past another person. First, in the process of walking past another person, it is necessary to understand the intention of the other person and predict what they may do next (Honma et al., 2015). Next, when an opponent is present in the direction of walk, individuals must use visual information and predict when and where a collision will occur (Patla, 1997; Gray and Regan, 2000). And then, based on accurately perceived visual information, they are required to avoid the opponent by modifying their movement pattern and making anticipatory gait adjustments (Higuchi, 2013). That is, in cooperative behavior with a partner and obstacle avoidance behavior, such as walking past someone, predicting and accurately perceiving changes in the external world underlies adaptive locomotor adjustments. Awkward gait characteristics associating with autistic traits while passing by another individual, as described by Shigeta et al. (2018), would be derived from the delayed and less sensitive visual processing of another person’s approach.

In this study, the N170 and P200 ERPs were identified for each participant in the AW and RW conditions. These two ERPs exhibited similar patterns in previous studies using optic flow as visual stimuli (Yamasaki et al., 2011). Consistent with the study, our results also showed that these ERPs are appropriate indicators to evaluate the visual processing associating with autistic traits. In addition, our results might support a previous study that suggested selective impairment in the dorsal pathway in visual information processing in individuals with diagnosis of ASD (Spencer et al., 2000; Yamasaki et al., 2011). Visual motion processing in humans involves two main pathways: the ventral and dorsal. The ventral visual pathway is involved in object and facial recognition. The dorsal visual pathway is primarily concerned with the spatial location of objects and motion stimuli (Vaina et al., 2001; Rizzolatti and Matelli, 2003). Visual processing of the approaching virtual avatar in this experiment suggests that the dorsal path is primarily used because it is necessary to understand the spatial position of the approaching avatar, although we could not confirm the idea since we did not conduct source localization. The previous study (Yamasaki and Tobimatsu, 2011) that used both of functional magnetic resonance imaging (fMRI) and ERP to demonstrate activation of brain regions to horizontal motion and radial optic flow. They revealed that N170 was evoked by both stimuli and originated from V5/MT, while P200 was evoked only in response to radial optic flow and originated from the inferior parietal lobule, a part of the ventrodorsal pathway. Furthermore, Yamasaki et al. (2011) showed that individuals with a diagnosis of ASD have delayed N170 and P200 latencies only to radial optic flow, and not to horizontal motion, suggesting an altered function of the higher-level dorsal visual stream in ASD. Thus, the delayed P200 in response to a human avatar’s approach in this study may be due to the a typicality of the ventrodorsal pathway in individuals with autistic traits. However, EEG measurements have lower spatial resolution compared to other measurement methods (e.g., fMRI). To clearly prove that the present delay in visual motion processing originates from the ventrodorsal pathway, additional measurements, such as fMRI, are needed in future studies.

Furthermore, “P200 latency in the AW condition” was the most frequently selected explanatory function in 36 of the 50 models, followed by “P200 amplitude in the AW condition,” which was selected in 28 models. “P200 amplitude in the RW condition” was the third-most frequently selected among the eight explanatory functions in 26 models. In other words, both the amplitude and latency of P200 in the AW condition were selected more than those in the RW condition at the T6 electrode. This may be because the visual stimulus in the AW condition was radial motion similar to radial optic flow showing expansion. In other words, the AW condition was perceived as an expanding motion when the virtual avatar was approaching. Individuals with autistic traits may have more specific visual processing for expanding movements (someone approaching them). In addition, the present results did not show a long latency or reduced amplitude of the N170 component. This result differs from those of a study using radial optic flow (Yamasaki et al., 2011) and PLW (Ichikawa et al., 2019; Inokuchi et al., 2022a) as the visual stimuli. This difference may be attributed to the fact that the visual stimulus is more natural and smoother in a virtual avatar video than the dot stimuli. The V5/MT in the brain region where the N170 component is evoked has been suggested to play a role in integrating the local motion signal from V1 and converting it into global motion (Snowden et al., 1991). However, because visual stimuli from a virtual avatar have already been converted to global biological motion, it is possible that longer latency and smaller amplitude were observed in P200 than those in N170.

Finally, three limitations should be taken into consideration when interpreting the current results. First, participants in this experiment were neurotypical university students, not diagnosed with ASD, and the variation in SATQ scores in this study was smaller than that in other ASD studies. Although the present study aimed to examine the association between autistic traits in neurotypical adults and the visual processing of an approaching life-sized avatar’s gait, the visual motion processing of individuals diagnosed with ASD could not be adequately verified. Second, only male participants were tested due to the nature of our faculty male-to-female ratio was not balanced (the ratio of men to women was 4 to 1). Future experiments with equal sex ratios may contribute to further clarification of autistic traits. Third, the spatial resolution of ERPs is far inferior to that of fMRI, and ERP measurements alone are limited in their spatial considerations of the brain. A more detailed spatial consideration of visual motion processing using fMRI is warranted.

## 5. Conclusion

In this study, we investigated the association between autistic traits in neurotypical male university students and the visual processing of another person’s approach by measuring the ERPs for videos of a life-sized virtual avatar performing approaching and retreating walking motions used as visual stimuli. As a result, the N170 and P200 for each participant at T5 and T6 electrodes were identified. Multiple regression analyses for each ERP indicator and cross-validation for each model were performed. The results showed that the P200 amplitude in the AW condition was the most selected variable for predicting the SATQ score at the T5 electrode, while the P200 latency in the AW condition was the most selected variable at the T6 electrode. Furthermore, the weight diagram results showed that the smaller the P200 amplitude at the T5 electrode and the longer the P200 latency at the T6 electrode in the AW condition, the higher SATQ score. These results suggest that the P200 latency observed at the T6 electrode and P200 amplitude observed at T5 and T6 electrodes in the AW condition explains the severity of autistic traits, and that individuals with autistic traits have delayed and less sensitive processing of the approach of others than those without autistic traits. Previous studies on autistic traits have focused on simplified visual stimuli, such as radial optic flow or PLW. Therefore, investigating the association between autistic traits and visual processing of another person’s approach using a more realistic life-sized virtual avatar may be useful to clarify the atypical gait traits of individuals with autistic traits in daily life. Future studies should focus on verifying the association between autistic traits and brain activity in exhaustive detail by measuring the ERPs during actual interactions involving people walking past one another.

## Data availability statement

The original contributions presented in this study are included in the article/Supplementary material, further inquiries can be directed to the corresponding author.

## Ethics statement

The studies involving human participants were reviewed and approved by the Ethics Committee of the Tokyo University of Science. The patients/participants provided their written informed consent to participate in this study.

## Author contributions

RI designed the study and performed the data analysis. RI, HI, MY, and HT contributed to programming, testing, data collection, and interpreted the data. All authors contributed to writing and editing of the manuscript and approved the final version of the manuscript for submission.

## Footnotes

1
https://sccn.ucsd.edu/eeglab/
